# Association of Over-The-Counter Pharmaceutical Sales with Influenza-Like-Illnesses to Patient Volume in an Urgent Care Setting

**DOI:** 10.1371/journal.pone.0059273

**Published:** 2013-03-21

**Authors:** Timothy Y. Liu, Jason L. Sanders, Fu-Chiang Tsui, Jessi U. Espino, Virginia M. Dato, Joe Suyama

**Affiliations:** 1 University of Pittsburgh, Pittsburgh, Pennsylvania, United States of America; 2 Real-time Outbreak and Disease Surveillance Laboratory, Pittsburgh, Pennsylvania, United States of America; 3 University of Pittsburgh Medical Center Urgent Care at Shadyside, Pittsburgh, Pennsylvania, United States of America; 4 Pennsylvania Department of Health, Harrisburg, Pennsylvania, United States of America; UC Davis School of Medicine, United States of America

## Abstract

We studied the association between OTC pharmaceutical sales and volume of patients with influenza-like-illnesses (ILI) at an urgent care center over one year. OTC pharmaceutical sales explain 36% of the variance in the patient volume, and each standard deviation increase is associated with 4.7 more patient visits to the urgent care center (p<0.0001). Cross-correlation function analysis demonstrated that OTC pharmaceutical sales are significantly associated with patient volume during non-flu season (p<0.0001), but only the sales of cough and cold (p<0.0001) and thermometer (p<0.0001) categories were significant during flu season with a lag of two and one days, respectively. Our study is the first study to demonstrate and measure the relationship between OTC pharmaceutical sales and urgent care center patient volume, and presents strong evidence that OTC sales predict urgent care center patient volume year round.

## Introduction

Surveillance and response to diseases are key priorities for the Centers for Disease Control and Prevention (CDC) and the U.S. public health system. [1] Syndromic surveillance is the real-time monitoring of health-related data for early detection of potential outbreaks before traditional, diagnosis-based surveillance by health departments. [2]–[4] Although initially developed to detect large-scale bioterrorism events, syndromic surveillance has the potential to rapidly and sensitively detect infectious disease outbreaks, enabling earlier response to minimize adverse impact. [5] Prior to confirmed medical diagnosis, behavioral patterns and non-clinical data of ailing patients may be analyzed and modeled from a variety of data sources [6].

One source of data for syndromic surveillance is over-the-counter (OTC) pharmaceutical sales. [7]–[8] OTC pharmaceuticals are purchased without a prescription and include cough syrup, flu remedies, or pediatric oral rehydration solutions. OTC sales have been correlated with infectious disease outbreaks. [9]–[12] Patients with influenza-like-illness (ILI) or upper respiratory tract infection (URI) are more likely to purchase OTC pharmaceuticals than present to their personal physician or an emergency department (ED). [9], [13] Furthermore, elevation in OTC pharmaceutical sales has been shown to precede elevation in ED or hospital patient volume by one to seventeen days. [10]–[12] Although these previous studies demonstrate that OTC sales correlate with ED and hospital patient volume for ILI and URI, most people with mild ILI or URI do not present initially to an ED or hospital. [14] These may be non-ideal settings to accurately track patient behavior and the underlying disease trends in a population, which is the goal of syndromic surveillance. Thus, these studies may have underestimated the power of OTC sales as a source of data for syndromic surveillance.

Urgent care centers provide walk-in ambulatory medical service for acute illness and injuries not serious enough to require the full resources of a hospital ED. [15] They are rapidly growing across the U.S. Since most cases at urgent care centers are related to respiratory illness, [16] correlating OTC sales to ILI patient volume at urgent care centers may more accurately illustrate the sensitivity of OTC sales as a syndromic monitoring method. In this analysis we examined the association between OTC sales and ILI patient volume at an urgent care center to determine the feasibility and functionality of OTC sales to monitor endemic ILI.

## Materials and Methods

IRB approval was obtained, and the authors have no conflicts of interests for this study.

### Measurement of OTC Pharmaceutical Sales

The National Retail Data Monitor (NRDM) is a public health surveillance tool that collects information on OTC health care products sold in over 29,000 stores across the country (rods.health.pitt.edu/NRDM.htm). The NRDM has been active since 2002. Sales data is updated electronically and stratified by zip codes, product sale category and promotion status. This system was pioneered by the Real-time Outbreak and Disease Surveillance Laboratory (RODS) at the Department of Biomedical Informatics at the University of Pittsburgh. [17]–[18] After approval from the Pennsylvania Department of Health, we used NRDM to tabulate daily quantities of OTC pharmaceuticals sold in thirteen zip codes comprising the greater Pittsburgh area from July 1, 2010 through July 31, 2011. Six out of eighteen sales categories from the NRDM system were selected for their relevance to ILI (Table 1). Each sales category was exclusive except for cough and cold, which is the sum of all sales of cold relief and cough products as well as additional uncategorized products. A previous study has demonstrated that the separation of products being on sale or sold at a discount does not affect the ability of the NRDM to distinguish disease activity in the community [18].

**Table 1:** Selected OTC Pharmaceutical Categories from the National Retail Data Monitor.

Categories	Used in analysis
Antidiarrheal	Not Selected
Antifever Pediatric	Selected
Antifever Adult	Selected
Bronchial Remedies	Not Selected
Chest Rubs	Not Selected
Cold Relief Adult Liquid	Not Selected
Cold Relief Adult Tablet	Not Selected
Cold Relief Pediatric Liquid	Not Selected
Cold Relief Pediatric Tablet	Not Selected
Cough Syrup Adult Liquid	Not Selected
Cough Adult Tablet	Not Selected
Cough Syrup Pediatric Liquid	Not Selected
Cough and Cold	Selected
Electrolytes Pediatric	Selected
Hydrocortisones	Not Selected
Nasal Product Internal	Not Selected
Thermometers	Selected
Throat Lozenges	Selected

OTC, over-the-counter.

### Urgent Care Overview

The UPMC Urgent Care at Shadyside (UPMC Urgent Care) is located in an urban neighborhood (zip code 15232) outside of Pittsburgh. It is owned and operated by UPMC, a large academic hospital system affiliated with the University of Pittsburgh School of Medicine. The UPMC Urgent Care opened in January 2010 with operating hours from 9 a.m. to 9 p.m. seven days per week. A physician is always present and is supported by additional mid-level providers as dictated by patient volume. The center employs between 3–5 nurses and ancillary staff. Most patients present to the UPMC Urgent Care for upper respiratory infection, flu-like symptoms, or minor trauma and lacerations. The UPMC Urgent Care utilizes an electronic medical record (EMR) from Epic (www.epic.com, Verona, Wisconsin) which is linked to the UPMC outpatient medical record system.

### Measurement of the Volume of Patients with ILI at the Urgent Care Center

Several methods have been used to detect an outbreak of ILI, including influenza surveillance data, manual review of hospital charts, and ICD-9 codes. [5] ICD-9 codes from the ED or hospitals have been used as the gold standard of patient volume in previous syndromic surveillance studies using OTC pharmaceutical sales. [10]–[12] For this study, daily patient volume of ILI at the UPMC Urgent Care was accessed from the Epic EMR system. Patients were selected on the basis of a set of 18 ICD-9 codes (Table 2) based on clinical knowledge and previous validations of similar code sets. [19]–[20] All data was de-identified and could not be linked back to any protected health information. This study was approved by the University of Pittsburgh Institutional Review Board as an exempt study.

**Table 2:** ICD-9 Code Set for the Selection of Patients with Influenza-Like-Illnesses.

Syndromes	ICD-9 Codes
Pharyngitis	462, 463, 034.0
URI	465.8, 465.9
Laryngitis	464.00, 464.4
Sinusitis	461, 461.9
Cough	786.2
Viral Syndrome	079.99
Pneumonia	486*
Otitis Media	382.9
Conjunctivitis	372
Bronchitis	490, 466
Flu	487.8, 487.1

* The documentation and coding system used at the Urgent Care system only included 486 as the diagnosis for all clinical pneumonia, as only clinical and radiographic evidence was available. No specific definitive testing was performed at the Urgent Care, and the only available ICD-9 code available for the provider to select at the time of disposition was 486 by default for any patient that qualified clinically for pneumonia.

### Statistical Analysis

Rather than use raw daily data, we decided *a priori* to use a 7-day moving-average (7dMA) for OTC sales and patient volume to minimize the day-of-the-week effect. [21]–[22] This is accomplished by averaging the variable over 3 days prior and 3 days after the day of interest. This dampens the day-of-the-week effect on OTC pharmaceutical sales and patient volume (i.e., reduces noise) while minimizing assumptions about data structure and other temporal trends.

In this analysis we examined the association of OTC sales (predictor) to urgent care ILI patient volume (outcome) using two analytic methods: 1) ordinary least squares regression (OLS); 2) autoregressive integrated moving average (ARIMA) modeling. OLS regression was used to determine the simple association of OTC sales to patient volume without taking into account the temporal nature of the data. This allows us to answer the questions: a) Is patient volume high when OTC sales are high? b) How many more patients present to urgent care with ILI, on average, with a standard increase in OTC sales? The shape of the association of OTC sales with patient volume was explored with lowess smoothed curves. Lowess curves suggested that over the majority of the data a linear trend was appropriate. Subsequently, OLS regression was used to determine the magnitude of the association of OTC sales with the volume of patients with ILI. We present the amount of variance in the volume of patients with ILI accounted for by OTC pharmaceutical sales with the each model’s overall r^2^. Model regression coefficients illustrate the average increase in number of patients presenting to urgent care with ILI given a standard deviation increase in OTC sales. This association was determined for the entire time period (July 1, 2010–July 31, 2011) and for pre-flu season (July 1, 2010–Nov 30, 2010), flu season (Dec 1, 2010–March 31, 2011), and post-flu season (April 1, 2011–July 31, 2011) to determine if OTC sales track with patient volume differently depending on the time of the year.

Next, we used ARIMA modeling to take into account the temporal nature of the data. ARIMA models calculate the correlation between two fluctuating signals, such as OTC sales and patient volume. Correlation is denoted by a cross correlation function (CCF). CCFs range from 0 (no correlation between signals) to 1 (perfect correlation between signals). CCFs are calculated for data on the same day and by shifting the signals in 1-day increments, called the lag. This allowed us to determine if OTC sales are most strongly correlated with patient volume on the same day, or if changes in OTC sales precedes changes in patient volume. Again, the association was determined for the entire time period and for pre-flu season, flu season, and post-flu season to determine if OTC sales track with patient volume differently depending on the time of the year.

For both OLS regression and ARIMA modeling we conducted a sensitivity analysis where we lengthened the flu season by one month (Dec 1, 2010–April 30, 2011). Trends were consistent with those from the primary analysis, so only results from the primary analysis are presented. For the entire analysis a two-sided p-value <0.05 was used to determine statistical significance. SAS 9.2 (SAS Institute Inc., Cary, NC) was used for all analysis.

## Results

**Figure 1:**
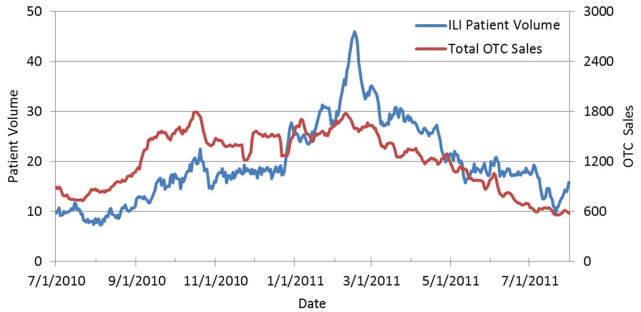
ILI Patient Volume vs. Total OTC Sales (7dMA). Seven day moving average (7dMA) of OTC pharmaceutical sales and volume of patients with influenza-like-illness (ILI) patient volume at the UPMC Urgent Care from July 1, 2010 to July 31, 2011. Volume of patients with ILI is blue and on the left y-axis, and OTC pharmaceutical sales is red and on the right y-axis.

**Figure 2:**
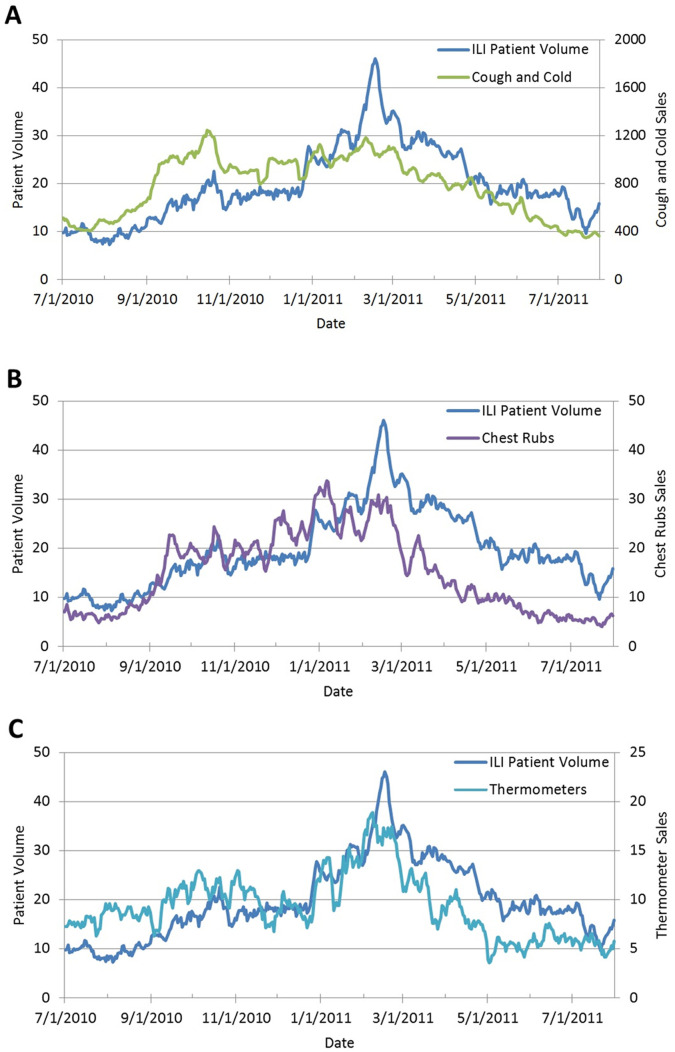
ILI Patient Volume vs. Cough and Cold, Chest Rub, and Thermometer Sales Volume (7dMA). Seven day moving average (7dMA) of sales volume of Cough and Cold medications (A), Chest Rubs (B), and Thermometers (C) in comparison with and volume of patients with influenza-like-illness (ILI) at the UPMC Urgent Care from July 1, 2010 to July 31, 2011. Volume of patients with ILI is blue and on the left y-axis, and the three different categories of OTC pharmaceutical sales are on the right y-axis.

Between July 1^st^, 2010 and July 31^st^, 2011, UPMC Urgent Care was visited by 7,813 patients with at least one of the 18 pre-selected ICD-9 codes (table 2). Among them, 68% of patients had only one ICD-9 diagnosis code, 27% had two codes, and 4% had three or more codes. The majority of the patients (66%) resided within the thirteen zip codes queried from the NRDM database. Of the thirteen zip codes requested, two did not have a pharmacy registered with the NRDM. Patients in those zip codes account for 6% (n = 470) of the sample and were included in the study. The 7dMA of total OTC pharmaceutical sales and ILI patient volume are shown in Figure 1. The 7dMA of the ILI patient volume against the sales of cough and cold, chest rubs, and thermometers are in Figure 2 (A, B, and C).

OLS regression indicated that sales of OTC pharmaceuticals were significantly and positively associated with ILI patient volume (Table 3). Over the entire time period, sales of cough and cold therapies, chest rubs, and thermometers were most strongly associated with ILI patient volume. Individually, these sales accounted for 36–39% of the variance in patient volume (P<0.0001). A SD increase in the sale of these pharmaceuticals was associated with an average increase of 4.8–4.9 patients presenting to urgent care with ILI. When the time period was stratified it was evident that associations between OTC sales and patient volume were influenced by time of the year. During pre-flu season, chest rubs, cough and cold therapies, and throat lozenges explained a large amount of variance in ILI patient volume (r^2^ = 0.771 to 0.829, all P<0.0001). Strikingly, during flu season, only thermometer sales was moderately associated with ILI patient volume (r^2^ = 0.485, P<0.0001), followed by cough and cold therapies (r^2^ = 0.164, P<0.0001). A SD increase in thermometer sales during flu season was associated with an average increase of 4.9 patients presenting with ILI to urgent care. In post-flu season, throat lozenges (r^2^ = 0.787, P<0.0001), antifever adult medicines (r^2^ = 0.708, P<0.0001), cough and cold therapies (r^2^ = 0.697, P<0.0001), and chest rubs (r^2^ = 0.662, P<0.0001) were most associated with ILI patient volume.

**Table 3:** Ordinary least square regression analysis of the association of OTC sales with urgent care ILI patient volume.

Entire time period (July 1, 2010–July 31, 2011)					
Category	r^2^	β*	SD (OTCsales)	B × SD**	P-value
Cough and Cold	0.385	0.020	245.5	4.9	<0.0001
Chest Rubs	0.366	0.591	8.1	4.8	<0.0001
Thermometers	0.363	1.485	3.2	4.8	<0.0001
Electrolytes Pediatric	0.269	0.952	4.3	4.1	<0.0001
Throat Lozenges	0.183	0.105	32.1	3.4	<0.0001
Antifever Adult	0.168	0.136	23.8	3.2	<0.0001
Antifever Pediatric	0.120	0.568	4.8	2.7	<0.0001
*Total*	*0.360*	*0.015*	*311.1*	4.7	*<0.0001*
**Pre-flu season (July 1, 2010–Nov 30, 2010)**					
**Category**	**r^2^**	**β** *	**SD (OTC** **sales)**	**β × SD** **	**P-value**
Cough and Cold	0.797	0.014	253.6	3.6	<0.0001
Chest Rubs	0.829	0.572	6.3	3.6	<0.0001
Thermometers	0.323	1.314	1.7	2.3	<0.0001
Electrolytes Pediatric	0.212	0.618	3.0	1.8	<0.0001
Throat Lozenges	0.771	0.138	25.3	3.5	<0.0001
Antifever Adult	0.465	0.248	11.0	2.7	<0.0001
Antifever Pediatric	0.083	0.427	2.7	1.1	0.0003
*Total*	*0.807*	*0.012*	*294.9*	*3.6*	*<0.0001*
**Flu season (Dec 1, 2010–March 31, 2011)**					
**Category**	**r^2^**	**β** *	**SD (OTC** **sales)**	**β × SD** **	**P-value**
Cough and Cold	0.164	0.032	89.5	2.8	<0.0001
Chest Rubs	0.003	0.071	5.2	0.4	0.56
Thermometers	0.485	1.540	3.2	4.9	<0.0001
Electrolytes Pediatric	0.000	0.040	3.1	0.1	0.85
Throat Lozenges	0.009	0.044	14.9	0.7	0.3
Antifever Adult	0.007	−0.042	13.7	−0.6	0.37
Antifever Pediatric	0.000	0.019	3.8	0.1	0.91
*Total*	*0.114*	*0.020*	*115.8*	2.3	*0.0002*
**Post-flu season (April 1, 2011–July 31, 2011)**					
**Category**	**r^2^**	**β** *	**SD (OTC** **sales)**	**β × SD** **	**P-value**
Cough and Cold	0.697	0.022	166.6	3.7	<0.0001
Chest Rubs	0.662	1.394	2.6	3.6	<0.0001
Thermometers	0.484	1.942	1.6	3.1	<0.0001
Electrolytes Pediatric	0.492	1.279	2.4	3.1	<0.0001
Throat Lozenges	0.787	0.185	21.0	3.9	<0.0001
Antifever Adult	0.708	0.155	23.8	3.7	<0.0001
Antifever Pediatric	0.529	1.389	2.3	3.2	<0.0001
*Total*	*0.723*	*0.017*	*216.7*	3.7	*<0.0001*

ILI, influenza like illness. OTC, over the counter medication. SD, standard deviation.

* β represents the increase in the number of urgent care patients per unit increase in the sale of OTC medication.

** β represents the increase in the number of urgent care patients per SD increase in the sale of OTC medication.

The ARIMA analysis was used to calculate the CCF between OTC sales and patient volume taking into account the temporality of the data. Overall, OTC sales and patient volume were significantly correlated (Table 4). Consistent with results from the OLS analysis, over the entire time period sales of cough and cold therapies (CCF = 0.621, P<0.05), chest rubs (CCF = 0.605, P<0.05), and thermometers (CCF = 0.602, P<0.05) were most strongly associated with patient volume. Stratification of the time period again indicated time-dependent associations between OTC sales and patient volume. In pre-flu season, chest rubs (CCF = 0.911, lag = 0 day, P<0.05), cough and cold therapies (CCF = 0.898, lag = 3 days, P<0.05), and throat lozenges (CCF = 0.878, lag = 0 days, P<0.05) were most strongly correlated with patient volume. Lags indicate that sale of cough and cold therapies was most strongly correlated with patient volume 3 days later, while sale of chest rubs and throat lozenges were most strongly correlated with patient volume on the same day. Notably, in pre-flu season, thermometer sales were strongly correlated with patient volume (CCF = 0.698, lag = 8 days, P<0.05), with a lag indicating that changes in thermometer sales preceded changes in patient volume by 8 days, a sizable amount.

**Table 4:** ARIMA analysis of the association of OTC sales with urgent care ILI patient volume.

Entire time period (July 1, 2010–July 31, 2011)			
Category	CCF (SE)	Lag*	P-value <0.05**
Cough and Cold	0.621 (0.050)	0	Y
Chest Rubs	0.605 (0.050)	0	Y
Thermometers	0.602 (0.050)	0	Y
Electrolytes Pediatric	0.519 (0.050)	0	Y
Throat Lozenges	0.428 (0.050)	0	Y
Antifever Adult	0.410 (0.050)	0	Y
Antifever Pediatric	0.347 (0.050)	0	Y
*Total*	*0.600 (0.050)*	*0*	*Y*
**Pre-flu season (July 1, 2010–Nov 30, 2010)**			
**Category**	**Maximum CCF (SE)**	**Lag** *	**P-value <0.05** **
Cough and Cold	0.898 (0.081)	3	Y
Chest Rubs	0.911 (0.081)	0	Y
Thermometers	0.698 (0.081)	8	Y
Electrolytes Pediatric	0.547 (0.081)	7	Y
Throat Lozenges	0.878 (0.081)	0	Y
Antifever Adult	0.715 (0.081)	−4	Y
Antifever Pediatric	0.481 (0.081)	15	Y
*Total*	*0.901 (0.081)*	*3*	*Y*
**Flu season (Dec 1, 2010–March 31, 2011)**			
**Category**	**Maximum CCF (SE)**	**Lag** *	**P-value <0.05** **
Cough and Cold	0.412 (0.091)	2	Y
Chest Rubs	−0.517 (0.091)	−15	Y
Thermometers	0.697 (0.091)	1	Y
Electrolytes Pediatric	0.293 (0.091)	9	Y
Throat Lozenges	0.568 (0.091)	−15	Y
Antifever Adult	0.135 (0.091)	14	N
Antifever Pediatric	0.323 (0.091)	15	Y
*Total*	*0.342 (0.091)*	*2*	*Y*
**Post-flu season (April 1, 2011–July 31, 2011)**			
**Category**	**Maximum CCF (SE)**	**Lag** *	**P-value <0.05** **
Cough and Cold	0.835 (0.092)	0	Y
Chest Rubs	0.813 (0.092)	0	Y
Thermometers	0.696 (0.092)	0	Y
Electrolytes Pediatric	0.701 (0.092)	0	Y
Throat Lozenges	0.887 (0.092)	0	Y
Antifever Adult	0.841 (0.092)	0	Y
Antifever Pediatric	0.727 (0.092)	1	Y
*Total*	*0.850 (0.092)*	*0*	*Y*

ARIMA, autoregressive integrated moving average. CCF, cross correlation function. ILI, influenza like illness. OTC, over the counter medication. SE, standard error.

* For analysis of the entire time period the lag was fixed to 0. For analysis of sub-periods the lag was limited to within 15 days. A positive lag indicates sale of OTC medication is correlated with future urgent care patient volume. A negative lag indicates sale of OTC medication is correlated with past urgent care patient volume.

** P-value <0.05 was determined if: CCF - (1.96×SE) >0.

During flu season thermometers were again most strongly associated with ILI patient volume (CCF = 0.697, lag = 1 day, P<0.05), with changes in sales preceding changes in patient volume by 1 day. It is notable though that the CCF for thermometers was highly stable across lags, indicating that thermometer sales have essentially equivalent correlation with ILI patient volume even using thermometer sales 1–7 days before patient volume. Cough and cold therapies were moderately correlated with patient volume (CCF = 0.412, lag = 2 days, P<0.05). Sale of chest rubs (CCF = −0.517, lag = −15 days, P<0.05) and throat lozenges (CCF = 0.568, lag = −15 days, P<0.05) were also moderately correlated with patient volume though the large negative lag indicates that fluctuations in patient volume preceded fluctuations in sale of these OTCs.

In post-flu season, throat lozenges, antifever adult medicine, cough and cold therapies, and chest rubs were most strongly correlated with patient volume, again consistent with results from OLS regression. Thermometers were less strongly correlated with patient volume but remained significant with a moderately high correlation (CCF = 0.696, lag = 0, P<0.05).

## Discussion

Syndromic surveillance is an important public health tool for the early detection and response of disease outbreaks, and its effectiveness depends on timeliness, sensitivity, and false-alarm rates. [5] OTC pharmaceutical sales have been used to predict patient volume using ED visits as a proxy of the underlying disease outbreak, [10]–[12] with the assumption that patients self-medicate with non-prescription products before presenting at the ED. [23] No studies have examined such relationship in an urgent care setting. This study found that there is strong association between changes in OTC pharmaceutical sales with patient visits for ILI symptoms at an academic-affiliated urgent care in an urban setting of Pittsburgh.

Results from our OLS regression and ARIMA models support the hypothesis that OTC sales are highly correlated with ILI patient volume at urgent care. Moreover, they suggest which OTCs are most correlated with patient volume at specific time points. The general trend between various OTC sales and ILI patient volume is that most OTC categories peaked before the flu season but thermometers peaked during the flu season (Figure 1 and 2). Specifically, during pre-flu and post-flu seasons, cough and cold therapies, chest rubs, and throat lozenges are most strongly associated with patient volume. During flu season, only sale of thermometers appeared strongly associated with ILI patient volume and the CCF was highly stable 1–7 days before patient volume. The purchasing behavior of consumers probably depends on the predominating symptoms, which include allergies or respiratory difficulties during the non-flu season and fever or cough during the flu season.

This study found strong independent associations between three categories of OTC pharmaceuticals collected by NRDM in the Pittsburgh region with the volume of patients with ILI at the UPMC Urgent Care at Shadyside. The increase in the sales of cough and cold, chest rubs, or thermometers explained 38%, 37%, and 36% of the variance in the volume of patients with ILI at the UPMC Urgent Care, respectively. For people with ILI symptoms, cough and cold medication can relieve rhinorrhea, coughing, or myalgia. Thermometers allow people to determine whether they have a fever. Even though chest rubs have questionable clinical evidence for symptom relief, there may be a local preference to this product in the Pittsburgh region. Of note, the r^2^ values from our analysis were lower than the results from previous studies on day-of-the-week effects (r^2^ = 0.36−0.38 vs. r^2^ = 0.75−0.86). [21]–[22] Part of the reason is because patient volume at the urgent care is known to be highly and independently dependent on day-of-the-week effects, while OTC pharmaceutical sales are a surrogate of disease outbreak which is independent of time on a daily scale.

During the flu season, more people purchase OTC pharmaceuticals and visit urgent cares as the prevalence of infectious diseases increases. Even though all NRDM categories were significantly related to volume of patients with ILI for the whole year in a regression analysis, we found that only the sales of thermometers and cough and cold medications were still significantly associated during the flu season. This is perhaps due to people purchasing all types of OTC pharmaceuticals and seeking medical attention at urgent cares for symptoms caused by non-influenza infections or allergic sources during the non-flu season. When contracted with influenza during the peak months, patients are more likely to have elevated temperature and develop symptoms needing thermometers and cough and cold medications. Specifically, the much higher variance of ILI patient volume explained by the thermometer sales over cough and cold sales (48.5% vs. 16.4%) perhaps is partly caused by the price difference and the specificity of fever in ILI. Moreover, results from our cross-correlation analysis indicated that surges in the volume of patients with ILI at the urgent care lagged behind changes in cough and cold medications by two days and behind thermometers by one day during the flu season. This suggests that people seek medical attention at urgent cares promptly after purchasing these OTC pharmaceuticals, as suggested by previous studies using patient visits at the ED [10]–[12].

The association between OTC pharmaceutical sales and volume of patients with ILI at the urgent care from our findings allows for another syndromic surveillance tool. Public health monitoring in an urgent care setting can be applicable for the surveillance for outbreaks of routine infectious diseases or uncommon but potentially devastating bioterrorism events. Infectious diseases accounted for only 2.6% of all ambulatory care visits and 11.2% of all ED visits, [24] but half of the visits to urgent cares. [16] Feasibility of urgent care for the detection of a bioterrorism event was examined at the Salt Lake 2002 Olympic Winter Games in Utah, where the state’s health departments collected electronic data from 19 urgent care facilities and found no unexpected or man-made disease outbreaks. [25] Because earlier intervention to prevent a bioterrorism event, even by several days, can potentially save thousands of lives and billions of dollars, [26] expanding syndromic surveillance at the urgent care setting could be cost effective and highly clinically relevant. De-identified urgent care data can be used throughout the whole year and augmented to include the sales of thermometers and cough and cold medications during flu season. Information can be channeled electronically to systems such as the National Electronic Disease Surveillance System and the Public Health Information Network, which provide Internet-based infrastructure and industry standards for interoperability and fast communication.

Another potential use of the syndromic surveillance from the NRDM data is to manage staffing at urgent cares to improve care delivery and infection control, [22] which observed that 18.5% less people left without being seen and 30% reduction in patient complaints when patient volume is predicted using non-clinical indicators such as day-of-the-week effects. As urgent cares see only 20–50 patients per day, [16] most places are not able to handle large fluctuations in patient volume. Predictors such as OTC pharmaceuticals can enhance the preparation of urgent cares to ensure that patients can receive proper treatment and thus control the spread of diseases, which is especially crucial during flu seasons. [27] Furthermore, longer wait times at the urgent cares may lead to increase in nosocomial infection as their equipment and personnel often harbor airborne infectious viruses [25].

One limitation of our study is that our data only spanned thirteen months, i.e. one flu season, and consisted of information at the Pittsburgh area. Therefore, our finding is more prone to fluctuations of purchasing pattern, medical attention seeking behavior, and disease outbreak of that year in the city that we examined. We suspected that by following the correlation of various OTC sales with ILI patient volume over multiple years would increase the power of the study but maintain the general pattern, as a study that spanned three years that observed OTC sales with ED patient volume have found [7].

### Conclusions

Our study is the first study to demonstrate and measure the relationship between OTC pharmaceutical sales and urgent care center patient volume, and presents strong evidence that OTC sales predict urgent care center patient volume year round. Larger studies using multiple sites across a longer period of time would allow for a more accurate analysis of the relationship between OTC pharmaceutical sales and volume of patients with ILI at the urgent cares.
